# Creatine in the Urine

**Published:** 1855-09

**Authors:** J. Cheston Morris

**Affiliations:** Philada.


### *Creatine in the Urine.*

By J. CHESTON MORRIS, M. D.

Though long acknowledged as a normal constituent of the urine, the first notice of creatine in pathological conditions appeared in the June No. of the Examiner, in a letter from Dr. Miltenberger of Baltimore to Dr. Neill of this city. That it should not have attracted earlier the attention of microscopists in urinary examinations, is easily accounted for by its ready solubility and the small proportion in which it exists. Liebig thought that the substances taken for lactic acid in the urine were in reality creatine and creatinine, (which differs mainly from the former in containing two equivalents less of water, and in its greater solubility,) and estimated them as constituting about 0.15 per cent. of normal urine—nearly equal to the quantity of uric acid. Like urea and uric acid, creatine is probably derived from the decomposition of muscular tissue: it is readily obtained from the juice of muscles, and has been detected in the blood by Robin and Verdeil. In cases therefore of muscular waste, nothing is more reasonable than to expect to find it in the urine. As it is soluble in 70 parts of cold and much less of boiling water, we cannot expect to meet with it deposited in fresh urine; but in evaporated urine I think I have met with it. In looking for it, the edges and corners of the cover-glass should be carefully examined, and, as Dr. Miltenberger suggests, after the specimen has been prepared for a day or two. The crystals are remarkably permanent, and correspond closely to the figures of creatine given in Funke's Atlas of Physiological Chemistry. It has been suggested that they might prove to have been cholesterin, or chloride of sodium; but their shape and appearance preclude the former doubt, while a drop of a solution of nitrate of silver instantly decides the latter. A granular brown matter takes the place of the crystals of chloride of sodium, while creatine remains nearly unaffected.

In recently examining the urine of B. P., a blacksmith, aged about 45, I found, after two days, crystals similar to those found by Dr. Miltenberger, (of which he has kindly furnished me a specimen,) situated in the position I have mentioned, viz., at the edge of the cover-glass. This man has suffered under the phosphatic diathesis for nearly three years, but has been relieved by the use of alkaline diluents. In this case we may, I think, attribute the presence of creatine to the incessant draught made upon his muscular system by his occupation.

We need more light on this subject from general experience, however, before any sound views of its pathology can be developed, or rational plan of treatment be deduced.

*Philada., Aug. 11th,* 1855.

